# Osteosarcopenia in Finland: prevalence and associated factors

**DOI:** 10.1007/s11657-024-01439-7

**Published:** 2024-08-30

**Authors:** Matias Blomqvist, Maria Nuotio, Katri Sääksjärvi, Seppo Koskinen, Sari Stenholm

**Affiliations:** 1 University of Turku, Turun Yliopisto, Turku, Finland; 2 Department of Geriatric Medicine, University of Turku, Turku, Finland; 3 Department of Geriatric Medicine, Turku University Hospital and University of Turku, Turku, Finland; 4 Population Health Unit, Finnish Institute for Health and Welfare, Helsinki, Finland; 5 Department of Public Health, University of Turku and Turku University Hospital, Turku, Finland; 6 Centre for Population Health Research, University of Turku and Turku University Hospital, Turku, Finland; 7 Research Services, Turku University Hospital and University of Turku, Turku, Finland

**Keywords:** Osteosarcopenia, Sarcopenia, Osteoporosis, Epidemiology, Prevalence, Older adults

## Abstract

**Summary:**

This cross-sectional study investigated osteosarcopenia prevalence and its correlates among 2506 adults aged 55 and older in Finland. Findings show 3.9% had osteosarcopenia, while 13.8% and 11.1% had probable sarcopenia only or osteoporosis only, respectively. Osteosarcopenia was associated with low BMI, impaired mobility, ADL limitations and depression. Sarcopenia appeared to drive these associations more than osteoporosis. Osteosarcopenia may be a risk factor for functional decline, hospitalization, and institutionalization, warranting further research.

**Purpose:**

Osteosarcopenia is a disorder consisting of concurrent osteoporosis and sarcopenia. This cross-sectional study using nationally representative data from Finland in 2000 aimed to determine the prevalence of osteosarcopenia in Finland. In addition, associations of sociodemographic, lifestyle, anthropometric, physical and mental function indicators, chronic conditions and various biomarkers with osteosarcopenia were examined.

**Methods:**

The study included 2506 subjects aged 55 and over (mean age 68.0 years, SD 9.0). Probable sarcopenia was defined as grip strength < 27 kg for men and < 16 kg for women. Osteoporosis was defined as either ultrasound-based bone density measurement of T < -2.5, or self-reported, pre-existing diagnosis of osteoporosis. Participants were categorized into 4 groups: no sarcopenia and no osteoporosis, probable sarcopenia only, osteoporosis only, and osteosarcopenia. Information on sociodemographic, lifestyle, anthropometric, physical and mental function indicators, chronic conditions and various biomarkers were collected via structured interview, questionnaires, clinical examination, and blood and urine samples.

**Results:**

The prevalence of probable sarcopenia, osteoporosis and osteosarcopenia was 13.8%, 11.1%, and 3.9%, respectively. Osteosarcopenia was associated with low BMI, slow gait speed, impaired mobility, impaired ability in the activities of daily living and depression. Of the two components, probable sarcopenia appeared to contribute to these associations more than osteoporosis.

**Conclusion:**

According to representative population-based study, about every fifth person with probable sarcopenia also has osteoporosis. Mobility and ADL limitations were more common among people with osteosarcopenia than those with osteoporosis or probable sarcopenia alone. Future studies are needed to examine osteosarcopenia as an independent risk factor for functional decline, hospitalization, and institutionalization.

**Supplementary Information:**

The online version contains supplementary material available at 10.1007/s11657-024-01439-7.

## Introduction

Sarcopenia is a muscle disorder characterized by loss of muscle mass and strength [1]. It is a risk factor for falls, fractures and functional decline and death [2–8]. Sarcopenia can develop slowly over the years as an individual ages without adequate physical exercise and nutrition, or rapidly due to an acute illness or injury that causes prolonged bedrest [1]. Prevalence of sarcopenia varies markedly between studies due to diverse definitions of the disorder [9]. According to the most recent version of the European Working Group on Sarcopenia in Older People (EWGSOP2), sarcopenia is probable if grip strength is low, and confirmed if also muscle mass is low [1]. Sarcopenia is considered severe if functional impairment, such as slow gait speed is also present [1].

Low bone mineral density (BMD) increases risk of fractures. A BMD of more than two and a half standard deviations below the mean value of a healthy young female population is considered osteoporosis [10].

Disorder of “sarco-osteopenia”, or “osteosarcopenia” has been suggested to be used in people with concurrent osteoporosis and sarcopenia [11]. The term osteosarcopenia is used to emphasize the role of sarcopenia in fragility fracture risk, and to promote consideration of sarcopenia in clinical decision making among frail adults [11]. Osteosarcopenia is unsurprisingly a risk factor for falls, fractures and mortality [12]. Sarcopenia has adverse effects on the bone due to lack of strengthening signals produced by biomechanical forces, and disrupted biochemical signalling between bone and muscle [13–15]. Although research on osteosarcopenia is still relatively scarce, better identification and characterization of this patient population could lead to better care of patients at risk of fragility fractures and functional decline.

The aim of this study was to determine the prevalence of osteosarcopenia in Finland by using a nationally representative sample of adults aged 55 years and older. In addition, the aim was to compare sociodemographic and anthropometric factors, physical and mental function, chronic illness, and various biomarkers between those with no sarcopenia and no osteoporosis, probable sarcopenia only, osteoporosis only, and osteosarcopenia.

## Methods

### Study population

This study is based on the Health 2000 survey, a nationwide health examination survey collecting information on the health, functional capacity and well-being of the Finnish population in 2000–2001. Ten thousand people aged 18 and older were selected randomly from the national population register, using a stratified two-stage cluster sampling design. To obtain a sufficient number of observations from the older population, the sampling fraction for people aged 80 and older was doubled. Community-dwelling as well as institutionalized people living in mainland Finland were included. The implementation of the Health 2000 survey has been reported elsewhere in more detail [16]. A total of 8028 subjects participated in the survey, of which 3439 were 55 years old or older. For this study, we included participants aged 55 years or older from whom information on sarcopenia and osteoporosis was available (*n* = 2506, 72.9%). To evaluate the representativeness of the final sample, we compared socioeconomic and lifestyle characteristics, as well as mobility limitations as a health indicator, between the final sample and all participants over 55 from the Health 2000 survey (Table 1).

**Table 1:** Characteristics of the study sample and Health 2000 Survey participants aged 55 years and older

	*Study Sample (n* = 2506*)*	*Health 2000 (n* = 3439*)*
Age, mean (SD)	68.0 (9.3)	70.1 (10.4)
Sex, Female, *n* (%)	1472 (58.7)	2081 (60.5%)
Education		
Highest, *n* (%)	374 (15.0)	431 (13.7)
Middle, *n* (%)	533 (21.4)	643 (20.4)
Lowest, *n* (%)	1590 (63.7)	2083 (66.0)
Married or cohabiting, *n* (%)	1503 (60.1)	1750 (55.0)
Physical activity		
Exercise training, *n* (%)	305 (12.7)	330 (12.1)
Active, *n* (%)	1398 (58.2)	1479 (54.2)
Inactive, *n* (%)	701 (29.2)	918 (33.7)
Smoking, Current or past, *n* (%)	981 (39.3)	1170 (38.7)
Mobility limitation, n (%)	661 (26.6)	1082 (34.3)

### Definition of probable sarcopenia

Grip strength was measured using an electronic device (Good Strength, IGS01, Metitur Oy, Finland) while the subject was sitting and resting their elbow on a table while gripping the handle of the device [16, 17]. The measurement was repeated after 30 s, and if there was a difference greater than 10%, a third measurement was taken. The highest result was ultimately used for analysis. Probable sarcopenia was defined according to the criterion by the EWGSOP2 [1]. Those with low grip strength were considered to have probable sarcopenia. Low grip strength was defined as < 27 kg for men and < 16 kg for women.

### Definition of osteoporosis

Bone mineral density was measured with a calcaneal ultrasound (Sahara Clinical Bone Sonometer, Hologic, Waltham, Massachusetts, USA). Quantitative ultrasound index (QUI) developed by the manufacturer was used as the measure of bone mineral density. QUI was derived from the speed of sound (SOS) and the broadband ultrasound attenuation (BUA) using the following formula:

$$QUI=0.41\times SOS+0.41\times BUA-571$$

Osteoporosis was defined as an ultrasound-based bone density measurement of T < -2.5. The reference group for *T*-score was 30–35-year-old women with no chronic illness or disability (*n* = 300) [10]. In addition, if the subject self-reported a pre-existing diagnosis of osteoporosis that was based on a DXA measurement (dual-energy X-ray absorptiometry) of the spine or hip, then the subject was considered to have osteoporosis regardless of their calcaneal ultrasound-based bone mineral density measurement. A total of 163 (8%) subjects self-reported a diagnosis of osteoporosis. Hip or spine was reported as the site of measurement for 41 (25%) subjects, while 7 (4%) reported measurement from the calcaneus, and 90 (54%) reported measurement from elsewhere. For 30 (18%) subjects, the site of measurement was not known.

### Osteosarcopenia

Osteosarcopenia was defined as concurrent probable sarcopenia and osteoporosis. Subjects were assigned into four groups: no sarcopenia and no osteoporosis, probable sarcopenia only, osteoporosis only, and osteosarcopenia.

### Measurement of other factors

Sociodemographic factors included age, sex, level of education (lowest, middle and highest) and marital status (married/cohabiting or living alone).

Information on lifestyle factors were obtained from survey questionnaires and included the level of physical activity (exercise training, active or inactive) [18], smoking (current/past or never), alcohol use (abstinence; low or medium use [< 140 g/week for women and < 280 g/week for men]; high use [≥ 140 g/week for women and ≥ 280 g/week for men] [19].

Dietary habits were assessed using a validated semi-quantitative 128-item food frequency questionnaire (FFQ) and the food consumption and nutrient intakes were calculated using the national food composition database (Fineli®) and in-house software [20, 21]. Variables included in this study were dairy product intake (g/d), protein intake (g/d), and total calcium intake from diet and supplements (g/d). The FFQ also contained additional questions about meal patterns and special diets, thus, variables on meal frequency (infrequent meals [fewer than 1–2 per day]; frequent meals [at least 1–2 times per day]), and lactose free diet (yes; no) were included. Anthropometric measurements included body mass index (BMI) based on body weight and height measurements, calculated as kg/m^2^, and self-reported weight loss during the previous 12 months. The data on weight was obtained using a bioimpedance measurement device InBody 3.0 (n = 2185, 87.2%), a digital scale (*n* = 122, 4.9%) or self-report (*n* = 196, 7.8%) if measurement was not available. Weight information was missing for 3 subjects. Height was measured for 2103 (83.9%) subjects and self-reported by 184 (7.3%) subjects. Height information was missing for 219 (8.7%) subjects. BMI was calculated as weight in kilograms divided by height in meters squared and further classified as underweight (< 18.5 kg/m^2^), normal weight (18.5–24.9 kg/m^2^), overweight (25–29.9 kg/m^2^) and obese (≥ 30 kg/m^2^).

At the health examination, maximal gait speed was measured over 6.1-m course [22]. In addition, subjects were asked “Can you walk 0.5 km without resting?” and “Can you climb up one flight of stairs without resting?” Subjects were considered to have mobility limitation if they reported any difficulties in walking 500 m or stair climbing. Similarly, questions were asked about performance in activities of daily living (ADL). “How do you manage the following activities (getting on or off the bed, getting clothes on or off, showering or using the bathroom)?” Subjects were considered to have ADL limitation if they reported difficulties in any of these activities.

Participants completed an abbreviated version of the Mini-Mental State Examination (MMSE) during an interview and total score was used in the analysis [23]. In addition, a question was asked on the depressiveness of their mood (feeling mostly sad or depressed on the day of the interview) [17].

Information on chronic conditions was based on self-reported diagnoses. Disease categories used in this study were diabetes, cancer excluding non-melanoma skin cancer, ocular disease (cataract, glaucoma, or retinal degeneration), hearing loss (hearing impairment of any type), psychiatric illness, arthrosis of the knee or the hip, pulmonary disease (asthma, chronic obstructive pulmonary disease, or chronic bronchitis), cardiovascular disease (CVD) or heart failure (myocardial infarction, angina pectoris, or heart failure), and stroke. Participants were also asked to evaluate their oral health condition and they were grouped as “good” and “other than good” (including somewhat good, average, somewhat poor and poor).

Fasting serum and spot urine samples were collected at the health examinations using standard procedures. In this study, we used information on serum vitamin-D-25, glycosylated hemoglobin-A1c (GHbA1c), serum creatinine, urine albumin, serum testosterone (males only), and C-reactive protein (CRP). Forced expiratory volume (FEV1) of a spirometry test was used to evaluate the function of the subject’s respiratory system. Detailed information about the measurements and classification are provided in the Supplementary information 1.

### Statistical analysis

The analysis was weighted to reduce bias due to nonresponse and to correct the oversampling in the age group of 80 years and older to represent the Finnish population. The complex sampling design was taken into account by using SAS survey procedures.

The Rao-Scott chi-square goodness-of-fit test was used to calculate prevalence of probable sarcopenia, osteoporosis and osteosarcopenia (SURVEYFREQ procedure in SAS), and all frequencies were weight-adjusted. Statistical differences between osteosarcopenia groups were tested using analysis of variance for normally distributed continuous variables, and Kruskal–Wallis test was used for continuous variables with skewed distributions (Serum CRP and urine album). A chi-squared test was used for categorical variables. Associations of categorical variables and osteosarcopenia groups were examined with logistic regression analysis for dichotomous variables and multinomial logistic regression analysis for three-level variables (SURVEYLOGISTIC procedure in SAS). Participants without osteoporosis and sarcopenia served as the reference group. In addition, to examine differences between the osteosarcopenia group and other groups, the osteosarcopenia group was used as the reference group. Associations between continuous variables and osteosarcopenia groups were examined with analysis of variance (GLM procedure in SAS). All analyses were initially adjusted for age and sex and then additionally for education and smoking, which are known correlates of sarcopenia and osteoporosis. To correct for the increased risk of Type I errors due to multiple comparisons, the Bonferroni correction was applied. Results that remained significant after applying the Bonferroni correction are indicated separately in Table 3, Table S1 and Table S2. The analyses were performed using SAS software version 9.4 (SAS Institute Inc., Cary, North Carolina, USA).

## Results

The current study sample was slightly younger and included more men and married/cohabiting participants than the entire Health 2000 survey participants aged 55 years and older (Table 1). In addition, the participants of the current study were physically more active (71% vs. 66%) and had less mobility limitations (27% vs. 34%) compared to all Health 2000 survey participants, but overall the differences were relatively small (Table 1).

The characteristics of the total study population and by osteosarcopenia groups are shown in Table 2. Overall, the mean age was 68.0 years (SD 9.0) and 57% of the subjects were female. The prevalences were the following: probable sarcopenia 13.8% (95% confidence interval [CI] 12.7–15.0%), osteoporosis 11.1% (9.9–12.3%) and osteosarcopenia 3.9% (3.2–4.6%). The mean age was highest in the osteosarcopenia group 78.0 years (SD 7.8) and the proportion of women was highest in the osteoporosis (89%) and osteosarcopenia (87%) groups. Being a current or past smoker was most common in the no sarcopenia and no osteoporosis group (44%) and the probable sarcopenia group (43%).

**Table 2:** Descriptive characteristics of the study sample by osteosarcopenia group

	*Overall*	*No sarcopenia, no osteoporosis*	*Sarcopenia only*	*Osteoporosis only*	*Osteosarcopenia*	*p*-value
*Weighted number of subjects*	2457 (100%)	1999 (81.4%)	200 (8.1%)	167 (6.8%)	89 (3.6%)	
*Sosiodemographic factors*						
*Age, mean (SD)*	68.0 (9.0)	65.3 (7.6)	73.1 (8.6)	70.9 (8.1)	78.0 (7.8)	< 0.001
*Sex, Female, n (%)*	1399 (57.0)	1046 (52.3)	127 (63.5)	148 (88.9)	77 (86.4)	< 0.001
*Education*						
*Highest, n (%)*	378 (15.5)	329 (16.6)	22 (11.2)	19 (11.5)	7 (8.3)	< 0.001
*Middle, n (%)*	535 (21.9)	444 (22.3)	31 (15.8)	44 (26.6)	15 (16.9)	
*Lowest, n (%)*	1534 (62.7)	1218 (61.1)	146 (73.0)	103 (61.9)	66 (74.8)	
*Fix intendation*	1534 (62.7)	1344 (67.5)	92 (46.0)	83 (50.0)	15 (17.4)	< 0.001
*Lifestyle factors*						
*Physical activity*						
*Exercise training, n (%)*	318 (13.4)	286 (14.7)	11 (6.3)	18 (11.2)	2 (3.3)	< 0.001
*Active, n (%)*	1420 (59.9)	1221 (62.6)	86 (47.4)	89 (55.4)	23 (31.3)	
*Inactive, n (%)*	632 (26.7)	444 (22.8)	84 (46.4)	53 (33.4)	49 (65.4)	
*Smoking, Current or past, n (%)*	998 (40.8)	849 (42.6)	84 (42.3)	40 (24.6)	23 (26.6)	< 0.001
*Alcohol*						
*Abstinence, n (%)*	751 (31.8)	541 (27.9)	89 (48.1)	72 (46.2)	47 (61.9)	< 0.001
*Medium or low use, n (%)*	1516 (64.1)	1320 (67.9)	91 (49.0)	77 (49.0)	27 (35.5)	
*High use, n (%)*	98 (4.1)	82 (4.3)	5 (2.9)	7 (4.8)	2 (2.6)	
*Infrequent meals (fewer than 1–2 per day) vs. frequent meals, n (%)*	654 (26.6)	562 (28.2)	40 (20.1)	31 (18.9)	19 (22.1)	0.005
*Lactose free diet, n (%)*	219 (10.1)	173 (9.6)	12 (8.3)	24 (18)	8 (12.6)	0.01
*Dairy product intake, g/d, mean (SD)*	628 (353)	621 (360)	689 (388)	622 (319)	640 (322)	0.40
*Protein intake, g/d, mean (SD)*	94 (36)	94 (35)	101 (43)	93 (39)	88 (29)	0.008
*Calcium intake, mg/d, mean (SD)*	1518 (627)	1508 (648)	1510 (598)	1609 (624)	1509 (498)	0.69
*Anthropometric measurements*						
*Body mass index (kg/m*^*2*^*)*						
*Underweight (*< *18.5), n (%)*	21 (0.9)	6 (0.3)	3 (1.9)	3 (2.3)	7 (8.6)	< 0.001
*Normal weight (18.5–24.9), n (%)*	670 (27.3)	509 (25.5)	62 (31.3)	59 (35.6)	37 (42.1)	
*Overweight (25–29.9), n (%)*	1067 (43.5)	903 (45.2)	69 (34.7)	66 (39.5)	28 (31.9)	
*Obese (*≥ *30), n (%)*	695 (28.3)	577 (28.9)	64 (32.1)	37 (22.6)	15 (17.4)	
*Weight loss*						
> *5 kg unintentionally vs. No weight loss, n (%)*	115 (4.9)	80 (4.2)	18 (10)	11 (7.6)	3 (5.4)	0.008
> *5 kg intentionally vs. No weight loss, n (%)*	109 (4.7)	95 (5.9)	7 (3.8)	6 (4.1)	1 (1.5)	
*1–5 kg vs. No weight loss, n (%)*	319 (13.7)	251 (13.0)	31 (17.2)	22 (14.4)	13 (19.2)	
*Physical and mental function*						
*Mobility limitation (500 m or stairs), n (%)*	573 (23.5)	336 (16.9)	103 (52.0)	65 (39.9)	67 (77.9)	< 0.001
*ADL limitation, n (%)*	323 (13.2)	166 (8.3)	73 (36.7)	35 (21.5)	48 (54.4)	< 0.001
*Depressive mood, n (%)*	846 (36.4)	662 (34.5)	85 (48.3)	55 (35.2)	43 (59.2)	< 0.001
*Grip strength, kg, mean (SD)*	31.1 (12.6)	34.5 (11.7)	15.7 (5.4)	25.2 (7.7)	13.1 (4.6)	< 0.001
*Gait speed, m/s, mean (SD)*	1.5 (0.4)	1.6 (0.4)	1.1 (0.4)	1.3 (0.3)	0.9 (0.3)	< 0.001
*FEV1 spirometry, L, mean (SD)*	2.5 (0.8)	2.6 (0.8)	2.1 (0.6)	2.2 (0.7)	1.6 (0.5)	< 0.001
*Short MMSE, median (Q1, Q3)*	14 (12, 15)	14 (13, 15)	13 (11, 15)	14 (12, 15)	13 (10, 15)	< 0.001
*Chronic conditions*						
*Diabetes, n (%)*	237 (9.7)	180 (9.1)	32 (16.3)	14 (8.9)	9 (10.7)	0.011
*Cancer excluding non-melanoma skin cancer, n (%)*	200 (8.2)	151 (7.6)	19 (9.6)	22 (13.7)	7 (8.1)	0.04
*Ocular disease, n (%)*	574 (23.4)	384 (19.2)	91 (45.5)	51 (30.9)	47 (52.5)	< 0.001
*Hearing loss, n (%)*	616 (25.2)	488 (24.5)	53 (26.5)	44 (26.4)	30 (33.8)	0.36
*Psychiatric illness, n (%)*	343 (14.0)	266 (13.4)	33 (16.7)	23 (13.9)	20 (22.7)	0.059
*Arthrosis of the knee or the hip, n (%)*	947 (38.7)	716 (36.0)	98 (49.3)	84 (50.6)	47 (54.0)	< 0.001
*Pulmonary disease, n (%)*	423 (17.3)	311 (15.6)	43 (21.9)	46 (27.7)	21 (25.0)	< 0.001
*CVD or heartfailure, n (%)*	521 (21.4)	367 (18.5)	69 (34.5)	44 (26.7)	40 (46.4)	< 0.001
*Stroke, n (%)*	118 (4.8)	82 (4.2)	18 (9.0)	9 (5.7)	8 (9.1)	0.004
*Oral health other than good, n (%)*	332 (13.6)	255 (12.8)	38 (19.4)	19 (11.8)	19 (21.4)	0.008
*Laboratory biomarkers*						
*Vitamin D-25, nmol/l, mean (SD)*	47 (18)	48 (17)	42 (17)	47 (21)	38 (14)	< 0.001
*GHbA1c, %, mean (SD)*	5.6 (0.8)	5.6 (0.8)	5.7 (0.9)	5.5 (0.6)	5.6 (0.8)	0.014
*Creatinine, µmol/l, mean (SD)*	75 (18)	75 (16)	78 (21)	73 (23)	72 (22)	< 0.001
*Urine albumin, mg/l, median (Q1, Q3)*	3.6 (0, 9.0)	3.4 (0, 8.0)	5.9 (0, 16.7)	4.6 (0, 11.5)	7.1 (3.8, 19.3)	< 0.001
*Testosterone (males), nmol/l, mean (SD)*	15 (6)	15 (6)	16 (7)	18 (8)	12 (6)	0.091
*CRP, mg/l, median (Q1, Q3)*	1.1 (0.4, 2.7)	1.1 (0.4, 2.5)	1.6 (0.7, 4.3)	1.1 (0.4, 2.9)	1.2 (0.6, 3.1)	< 0.001

### Sociodemographic and lifestyle factors

Compared to the no sarcopenia, no osteoporosis group, subjects in the osteosarcopenia and probable sarcopenia only groups were more likely to be living alone and physically inactive (Table 3). High alcohol use was more common in the osteoporosis group compared to the no sarcopenia, no osteoporosis group (OR 3.11, 95% CI 1.33 – 7.27), but was no longer statistically significant after Bonferroni correction for multiple comparison. When compared to the osteosarcopenia group, the osteoporosis only group had higher involvement in exercise training (OR 4.25, 95% CI 1.04 – 17.41) and being physically active (OR 2.39, 95% CI 1.31 – 4.35). (Table 3, Table S1).

**Table 3:** The association of sociodemographic and lifestyle factors, and physical and mental conditions with osteosarcopenia group

	*No sarcopenia, no osteoporosis*	*Probable sarcopenia only*	*Osteoporosis only*	*Osteosarcopenia*
*Sosiodemographic factors*	*OR. Reference*	*OR (95% CI)*	*OR (95% CI)*	*OR (95% CI)*
*Married or cohabiting vs. Living alone*	1.00	0.68 (0.50 - 0.94)	0.96 (0.67 - 1.39)	0.27 (0.15 - 0.50)^b^
*Lifestyle factors*				
*Physical activity*				
*Exercise training vs. Inactive*	1.00	0.36 (0.19 - 0.68)	0.95 (0.52 - 1.74)	0.22 (0.06 - 0.83)
*Active vs. Inactive*	1.00	0.55 (0.40 - 0.75)^b^	0.79 (0.55 - 1.14)	0.33 (0.20 - 0.55)^b^
*Alcohol*				
*High use vs. Abstinence*	1.00	0.99 (0.39 - 2.54)	3.11 (1.33 - 7.27)	3.37 (0.78 - 14.53)
*Medium or low use vs. Abstinence*	1.00	0.69 (0.49 - 0.96)	0.80 (0.57 - 1.13)	0.71 (0.43 - 1.16)
*Infrequent meals (fewer than 1–2 per day) vs. frequent meals*	1.00	0.78 (0.53 - 1.14)	0.67 (0.45 - 1.02)	1.00 (0.59 - 1.70)
*Lactose free diet*	1.00	0.85 (0.44 - 1.66)	1.92 (1.15 - 3.21)	1.34 (0.64 - 2.82)
	*Adjusted mean (95% CI)*	*Adjusted mean (95% CI)*	*Adjusted mean (95% CI)*	*Adjusted mean (95% CI)*
*Dairy product intake, g/d*	635 (618 - 652)	638 (584 - 693)	592 (532 - 651)	526 (450 - 603)
*Protein intake, g/d*	95 (93 - 96)	99 (93 - 104)	91 (85 - 97)	83 (75 - 91)
*Calcium intake, mg/d*	1467 (1259 - 1676)	1467 (1259 - 1676)	1503 (1311 - 1696)	1356 (1091 - 1620)
*Anthropometric measurements*				
*Body mass index (kg/m*^*2*^*)*	*OR, Reference*	*OR (95% CI)*	*OR (95% CI)*	*OR (95% CI)*
*Underweight (*< *18.5) vs. Normal weight (18.5–25)*	1.00	4.89 (1.14 - 20.90)	4.66 (1.20 - 18.14)	10.56 (2.65 - 42.14)^b^
*Overweight (25–29.9) vs. Normal weight (18.5–25)*	1.00	0.61 (0.42 - 0.88)	0.59 (0.40 - 0.87)	0.39 (0.24 - 0.63)^b^
*Obese (*≥ *30) vs. Normal weight (18.5–25)*	1.00	0.89 (0.61 - 1.29)	0.47 (0.30 - 0.73)^b^	0.32 (0.18 - 0.57)^b^
*Weight loss*				
> *5 kg unintentionally vs. No weight loss*	1.00	1.93 (1.11 -3.36)	1.55 (0.79 - 3.04)	0.84 (0.34 - 2.10)
> *5 kg intentionally vs. No weight loss*	1.00	1.24 (0.52 -2.96)	1.13 (0.47 - 2.72)	0.53 (0.06 - 5.01)
*1–5 kg vs. No weight loss*	1.00	1.23 (0.82 - 1.84)	0.90 (0.57 - 1.45)	0.96 (0.51 - 1.81)
*Physical and mental function*	*OR, Reference*	*OR (95% CI)*	*OR (95% CI)*	*OR (95% CI)*
*Mobility limitation*	1.00	2.95 (2.10 - 4.15)^b^	2.01 (1.40 - 2.90)^b^	6.42 (3.59 - 11.50)^b^
*ADL limitation*	1.00	4.36 (3.19 - 5.94)^b^	2.55 (1.73 - 3.76)^b^	7.84 (4.89 - 12.58)^b^
*Depressive mood*	1.00	1.72 (1.25 - 2.38)^b^	0.97 (0.66 - 1.41)	2.75 (1.63 - 4.64)^b^
	*Adjusted mean (95% CI)*	*Adjusted mean (95% CI)*	*Adjusted mean (95% CI)*	*Adjusted mean (95% CI)*
*Grip strength, kg*	34.0 (33.7 - 34.3)	20.2 (19.4 - 21.0)^b^	32.8 (31.8 - 33.7)	23.2 (22.0 - 24.4)^b^
*Gait speed, m/s*	1.53 (1.51 - 1.54)	1.27 (1.23 - 1.32)^b^	1.43 (1.38 - 1.48)^b^	1.21 (1.15 - 1.28)^b^
*FEV1 spirometry, L*	2.55 (2.52 - 2.57)	2.39 (2.31 - 2.47)	2.58 (2.49 - 2.66)	2.37 (2.25 - 2.49)
*Short MMSE*	13.6 (13.5 - 3.7)	12.9 (12.6 - 13.2)^b^	13.2 (12.9 - 13.5)	12.6 (12.2 - 13.1)^b^
*Chronic conditions*	*OR, Reference*	*OR (95% CI)*	*OR (95% CI)*	*OR (95% CI)*
*Diabetes*	1.00	1.59 (1.05 - 2.39)	0.90 (0.48 - 1.67)	0.95 (0.48 - 1.88)
*Cancer excluding non-melanoma skin cancer*	1.00	0.83 (0.49 - 1.40)	1.27 (0.81 - 2.00)	0.57 (0.30 - 1.12)
*Ocular disease*	1.00	1.69 (1.20 - 2.37)	0.88 (0.59 - 1.31)	1.34 (0.83 - 2.15)
*Hearing loss*	1.00	0.82 (0.58 - 1.17)	1.11 (0.77 - 1.59)	1.04 (0.62 - 1.76)
*Psychiatric illness*	1.00	1.49 (0.99 - 2.22)	1.07 (0.63 - 1.81)	2.42 (1.38 - 4.24)
*Arthrosis of the knee or the hip*	1.00	1.58 (1.17 - 2.15)	1.54 (1.11 - 2.14)	1.73 (1.14 - 2.65)
*Pulmonary disease*	1.00	1.43 (0.99 - 2.06)	1.95 (1.34 - 2.84)^b^	1.77 (1.02 - 3.07)
*CVD or heartfailure*	1.00	1.41 (1.03 - 1.94)	1.16 (0.78 - 1.73)	1.87 (1.22 - 2.87)
*Stroke*	1.00	1.66 (1.04 - 2.63)	1.38 (0.72 - 2.63)	1.71 (0.79 - 3.71)
*Oral health other than good*	1.00	1.48 (1.02 - 2.15)	0.94 (0.59 - 1.52)	1.71 (0.99 - 2.95)
*Laboratory biomarkers*	*Adjusted mean (95% CI)*	*Adjusted mean (95% CI)*	*Adjusted mean (95% CI)*	*Adjusted mean (95% CI)*
*Vitamin D-25, nmol/l*	47.3 (46.5 - 48.1)	43.6 (41.2 - 46.0)	47.8 (45.1 - 50.6)	40.8 (37.1 - 44.5)
*GhbA1c, %*	5.60 (5.56 - 5.63)	5.64 (5.54 - 5.74)	5.48 (5.36 - 5.60)	5.47 (5.31 - 5.62)
*Creatinine, µmol/l*	77.2 (76.4 - 78.0)	77.1 (74.8 - 79.4)	76.9 (74.3 - 79.5)	73.0 (69.5 - 76.4)
*Urine albumin, Log(mg/l)*	2.44 (2.37 - 2.52)	2.63 (2.44 - 2.83)	2.61 (2.38 - 2.85)	2.29 (2.02 - 2.56)
*Testosterone (males), nmol/l*	15.2 (14.8 - 15.6)	15.9 (14.5 - 17.3)	18.2 (15.5 - 21.0)	12.3 (8.5 - 16.0)
*CRP, Log(mg/l)*	0.14 (0.07 - 0.20)	0.59 (0.40 - 0.78)	0.12 (-0.10 - 0.33)	0.30 (0.02 - 0.59)

Results are presented for categorical variables as odds ratios (OR) and mean values for continues variables, and their 95% confidence intervals (CI). Analyses are adjusted for age, sex, education and smoking. ^b^Statistically significant difference compared to the no sarcopenia, no osteoporosis group after Bonferroni correction

In terms of diet, adhering to a lactose-free diet was more common (OR 1.92, 95% CI 1.15 – 3.21) in the osteoporosis only group compared to the no sarcopenia, no osteoporosis group (OR 0.65, 95% CI 0.42 – 0.99; OR 2.14, 95% CI 1.27 –3.63; respectively). (Table 3) Protein and dairy intake were significantly lower in the osteosarcopenia group compared to the no sarcopenia, no osteoporosis group (83 g/d and 526 g/d vs. 95 g/d and 635 g/d, respectively), whereas in the probable sarcopenia only and osteoporosis only groups protein or dairy intake was similar to that in the no sarcopenia, no osteoporosis group. These differences were however not significant after applying Bonferroni correction for multiple comparisons. No difference was observed between probable sarcopenia or osteoporosis group and osteosarcopenia group. (Table S1). There were no significant differences in calcium intake or meal frequency between the groups. (Table 3).

### Anthropometric measures

Being underweight was more common in both the probable sarcopenia only, osteoporosis only and osteosarcopenia groups compared to the no sarcopenia, no osteoporosis group, and the odds were highest in the osteosarcopenia group (OR 10.56, 95% CI 2.65 – 42.14). The odds of being obese were significantly lower in the osteoporosis only and osteosarcopenia groups, but not in the probable sarcopenia only group, compared to the no sarcopenia, no osteoporosis group. In terms of weight loss, the probable sarcopenia only group was the only group showing higher odds for unintentional weight loss (OR 1.93, 95% CI 1.11 – 3.36) compared to the no sarcopenia, no osteoporosis group, but this was not statistically significant after applying Bonferroni correction for multiple comparisons.(Table 3). No difference in anthropometric measures were observed between probable sarcopenia or osteoporosis group and osteosarcopenia group, except that overweight and obesity were more common in probable sarcopenia only group compared to osteosarcopenia group (Table S1).

### Physical and mental function

Self-reported mobility and ADL limitations were more common in the probable sarcopenia only (OR 2.95 95% CI 2.10 – 4.15), osteoporosis only (OR 2.01, 95% CI 1.40 – 2.90) and osteosarcopenia (OR 6.42, 95% CI 3.59 – 11.50) groups compared to the no sarcopenia, no osteoporosis group (Table 3). When compared to the osteosarcopenia group, probable sarcopenia only and osteoporosis only groups had significantly lower odds ratios in both mobility and ADL limitation; however, after applying the Bonferroni correction, the difference between the osteosarcopenia and probable sarcopenia groups was not statistically significant (Table S1). Hand grip strength and gait speed were significantly lower/slower in the probable sarcopenia only and osteosarcopenia groups compared to the no sarcopenia, no osteoporosis group and osteoporosis only group. The probable sarcopenia only and osteosarcopenia groups had lower lung capacity measured with FEV1 than other groups, but no longer statistically significant after Bonferroni correction (Table 3).

Depressive mood was more common and MMSE points lower in the probable sarcopenia only and osteosarcopenia groups, but not in the osteoporosis only group compared to the no sarcopenia, no osteoporosis group (Table 3). Depressive mood did not differ between probable sarcopenia only and osteosarcopenia groups (Table S1).

### Chronic conditions

 Compared with the no sarcopenia, no osteoporosis group, the odds of knee and hip osteoarthrosis were elevated in the probable sarcopenia only, osteoporosis only, and osteosarcopenia groups (Table 3). However, the odds of osteoarthrosis did not differ significantly between these three groups (Table S1).Several conditions were associated specifically with sarcopenia. The probable sarcopenia only and osteosarcopenia groups both had higher odds of cardiovascular disease or heart failure (OR 1.41, 95% CI 1.03–1.94 and OR 1.87, 95% CI 1.22–2.87, respectively) (Table 3). The probable sarcopenia only group also exhibited a greater prevalence of stroke (OR 1.66, 95% CI 1.04–2.63), ocular disease (OR 1.69, 95% CI 1.20–2.37), and diabetes (OR 1.59, 95% CI 1.05–2.39) compared to the no sarcopenia, no osteoporosis group. Similarly, poor oral health was more common in the probable sarcopenia only group (OR 1.48, 95% CI 1.02–2.15) and showed a non-significant trend in the osteosarcopenia group (OR 1.71, 95% CI 0.99–2.95) compared to the no sarcopenia, no osteoporosis group.The osteosarcopenia group was uniquely associated with a higher prevalence of psychiatric illness, both when compared to the no sarcopenia, no osteoporosis group (Table 3) and the osteoporosis only group (Table S1). No significant differences were observed across the four groups for cancer or hearing loss (Table 3).After applying the Bonferroni correction for multiple comparisons, the only association that remained statistically significant was the higher odds of pulmonary diseases in the osteoporosis only group compared to the no sarcopenia, no osteoporosis group.

### Laboratory tests

As an indicator of chronic inflammation, CPR was elevated in the probable sarcopenia only and osteosarcopenia groups and was higher in the probable sarcopenia only group compared to osteosarcopenia group. The probable sarcopenia only and osteosarcopenia groups had comparable and lower levels of serum vitamin-D levels than other groups. These differences however did not remain significant after applying the Bonferroni correction. There were no differences between groups in GHbA1c, creatinine, testosterone, or urine albumin across osteosarcopenia groups (Table 3).

## Discussion

The prevalence of probable sarcopenia, osteoporosis, and osteosarcopenia in a representative population-based sample of Finnish adults was 13.8%, 11.1%, and 3.9%, respectively. Our study revealed that osteosarcopenia was associated with factors related to frailty, including impaired mobility, low bodyweight and depression. Physical performance was impaired and relatively similar among those with osteosarcopenia and probable sarcopenia only, but clearly different from those with osteoporosis only or neither sarcopenia nor osteoporosis. Mobility and ADL limitations were more common in the osteosarcopenia group than in the probable sarcopenia or osteoporosis only groups.

Previous studies have reported varying overall prevalence rates of osteosarcopenia, ranging from 5 to 37% [24]. However, these studies often had older study populations, a higher proportion of women, and some samples were specifically selected from populations with a history of falling. Additionally, differences in methodology, definitions, and genetic, environmental, and cultural factors in the studied populations may contribute to variations in results.

There are currently no studies on osteoporosis prevalence in Finland based on WHO guidelines and DXA-based BMD measurement. In the USA, the unadjusted prevalence of osteoporosis in people aged 65 and older was 5.1% for men and 24.5% for women between 2005 and 2010 [25]. Similarly, a Swedish study found the prevalence to be 21.2% in women and 6.3% in men aged 50–84 [26]. In our study, we adopted a *T*-score of − 2.5 as the threshold for diagnosing osteoporosis, based on the World Health Organization (WHO) definition [10]. Although using calcaneal ultrasound, a study by López-Rodrígues (2003) suggested that a *T*-score of − 1.5 provides the best balance between sensitivity and specificity in diagnosing osteoporosis compared to the gold standard dual-energy X-ray absorptiometry (DXA) method [27]. Therefore, it is possible that our results may underestimate the prevalence of osteoporosis. The clinical importance of our study is to identify potential conditions associated with an increased risk of fracture and other hazards, thereby adding value to existing risk assessment tools. Therefore, based on the expected prevalence from previous studies and clinical reasoning, we chose to accept the potential underestimation of osteoporosis with a *T*-score threshold of − 2.5 to further accentuate the differences between the study groups (osteosarcopenia, probable sarcopenia only, osteoporosis only, and no sarcopenia or osteoporosis).

According to the 2019 European Working Group on Sarcopenia in Older People (EWGSOP2) statement, the assessment of sarcopenia involves evaluating both muscle strength and muscle mass. In our study, we utilized only grip strength, based on the EWGSOP2 criteria for probable sarcopenia. Consequently, the actual prevalence of sarcopenia may be lower than that which we found. However, it is important to note that diagnostic criteria for sarcopenia are continuously evolving, and current guidelines emphasize the significance of reduced muscle strength, especially in clinical practice [28].

Several studies have explored the association of osteosarcopenia with adverse health and functional outcomes, though methodologies and definitions vary markedly. In the study by Huo et al. (2015) involving patients referred to an Australian falls and fractures clinic, osteopenia was defined using a DXA-based BMD T-score lower than − 1.0 and sarcopenia was defined by having at least two of the following conditions: low gait speed, low grip strength and low appendicular lean mass measured by DXA. Notably, osteopenia and not osteoporosis was a component of osteosarcopenia in addition to sarcopenia. Okamura et al. (2020) conducted a retrospective study of patients treated in a Tokyo centre for osteoporosis. They defined osteosarcopenia as osteoporosis concurrent with sarcopenia and used DXA-based *T*-score lower than − 2.5 or a previous fragility fracture as definitive of osteoporosis. Sarcopenia was defined by low gait speed or both low muscle mass and low grip strength. A study by Reiss et al. (2019) studying geriatric inpatients considered sarcopenia to be present if appendicular muscle mass measured by DXA was low and either grip strength or gait speed was low. Osteoporosis was defined as having *T*-score lower than − 2.5 using DXA.

In line with our findings, previous studies have also reported associations of osteosarcopenia with low BMI, malnutrition, osteoarthritis, and depression. In addition, osteosarcopenia has been associated with impaired mobility and slower gait speed compared to having osteoporosis only [29–31]. In the study by Huo et al. (2015), no differences between osteosarcopenia and sarcopenia only groups were found in terms of mobility limitation or depression. In our study, which represents the general population, those with osteosarcopenia had higher odds of depression and impaired mobility compared to those with probable sarcopenia only. Our results contrasted with those of Huo et al. (2015) regarding the associations of osteosarcopenia with cancer, pulmonary disease, and heart disease. The difference in these results might be due to the inclusion criteria of having past falls or increased risk of fractures in the study of Huo et al., in addition to differences in methodology and definitions of osteosarcopenia, sarcopenia, impaired mobility and depression. Overall, our results suggest sarcopenia as making a greater contribution to factors of functional decline than osteoporosis.

This study benefits from a large nationwide sample that is representative of the Finnish general population. The extensive study population and the comprehensiveness of our study, which included health examinations, surveys, and laboratory tests, made the inclusion of numerous factors for analysis possible. The methods used to assess bone mineral density and grip strength are well established.

Some limitations should also be acknowledged. DXA-based assessments of muscle mass and bone mineral density were not available. Moreover, the study sample was slightly younger and better functioning compared to the nationally representative Health 2000 Survey participants, which may limit generalizability of the findings to general population. Furthermore, the data were collected in 2000, and the prevalence of sarcopenia, osteoporosis, and osteosarcopenia may have changed since then due to the aging population.

In conclusion, subjects with osteosarcopenia have more mobility and ADL limitations compared to those with either probable sarcopenia or osteoporosis only. The potential additional value of combining these two known risk factors to predict risk of fall-related injuries, decline in mobility, loss of ability to perform daily activities, and death compared to only having one component of osteosarcopenia must be validated in future longitudinal studies.

## Supplementary Information

Below is the link to the electronic supplementary material.

Supplementary file1 (PDF 566 KB)

Supplementary file2 (PDF 368 KB)

## Data Availability

The supporting data for this research can be obtained from the Finnish Institute for Health and Welfare with restrictions. There are limitations on accessing these data as they were utilized under a license for this specific study and are therefore not openly accessible. The authors can provide the data upon a justified request, subject to the approval of the Finnish Institute for Health and Welfare.

## References

[1] Cruz-Jentoft AJ, Bahat G, Bauer J et al (2019) Sarcopenia: revised European consensus on definition and diagnosis. Age Ageing 48(1):16–31. 10.1093/ageing/afy169 30312372 10.1093/ageing/afy169 PMC6322506

[2] Bischoff-Ferrari HA, Orav JE, Kanis JA et al (2015) Comparative performance of current definitions of sarcopenia against the prospective incidence of falls among community-dwelling seniors age 65 and older. Osteoporos Int 26(12):2793–2802. 10.1007/S00198-015-3194-Y 26068298 10.1007/s00198-015-3194-y

[3] Schaap LA, Van Schoor NM, Lips P, Visser M (2018) Associations of Sarcopenia definitions, and their components, with the incidence of recurrent falling and fractures: the longitudinal aging study Amsterdam. J Gerontol A Biol Sci Med Sci 73(9):1199–1204. 10.1093/GERONA/GLX245 29300839 10.1093/gerona/glx245

[4] Malmstrom TK, Miller DK, Simonsick EM et al (2016) SARC-F: a symptom score to predict persons with sarcopenia at risk for poor functional outcomes. J Cachexia Sarcopenia Muscle 7(1):28. 10.1002/JCSM.12048 27066316 10.1002/jcsm.12048 PMC4799853

[5] Celis-Morales CA, Petermann F, Hui L et al (2017) Associations between diabetes and both cardiovascular disease and all-cause mortality are modified by grip strength: evidence from UK Biobank, a prospective population-based cohort study. Diabetes Care 40(12):1710–1718. 10.2337/dc17-0921 28986505 10.2337/dc17-0921

[6] Bone AE, Hepgul N, Kon S, Maddocks M (2017) Sarcopenia and frailty in chronic respiratory disease. Chron Respir Dis 14(1):85–99. 10.1177/1479972316679664 27923981 10.1177/1479972316679664 PMC5720213

[7] Chang KV, Hsu TH, Wu WT, Huang KC, Han DS (2016) Association between sarcopenia and cognitive impairment: a systematic review and meta-analysis. J Am Med Dir Assoc 17(12):1164.e7-1164.e15. 10.1016/J.JAMDA.2016.09.013 27816484 10.1016/j.jamda.2016.09.013

[8] de Buyser SL, Petrovic M, Taes YE et al (2016) Validation of the FNIH sarcopenia criteria and SOF frailty index as predictors of long-term mortality in ambulatory older men. Age Ageing 45(5):603–609. 10.1093/AGEING/AFW071 10.1093/ageing/afw071 27126327

[9] Carvalho do Nascimento PR, Bilodeau M, Poitras S (2021) How do we define and measure sarcopenia? A meta-analysis of observational studies. Age Ageing:1–8. 10.1093/AGEING/AFAB148 10.1093/ageing/afab148 34537833

[10] World Health Organization (1994) Assessment of fracture risk and its application to screening for postmenopausal osteoporosis: report of a WHO study group [meeting held in Rome from 22 to 25 June 1992]. https://iris.who.int/handle/10665/39142. Accessed 21.8.2024 7941614

[11] Binkley N, Buehring B (2009) Beyond FRAX®: it’s time to consider “Sarco-Osteopenia.” J Clin Densitom 12(4):413–416. 10.1016/j.jocd.2009.06.004 19733110 10.1016/j.jocd.2009.06.004

[12] Teng Z, Zhu Y, Teng Y et al (2021) The analysis of osteosarcopenia as a risk factor for fractures, mortality, and falls. Osteoporos Int. 10.1007/s00198-021-05963-x 10.1007/s00198-021-05963-x 33877382

[13] Herrmann M, Engelke K, Ebert R et al (2020) Interactions between muscle and bone—where physics meets biology. Biomolecules 10(3):432. 10.3390/biom10030432 32164381 10.3390/biom10030432 PMC7175139

[14] Paintin J, Cooper C, Dennison E (2018) Osteosarcopenia. Br J Hosp Med 79(5):253–258. 10.12968/hmed.2018.79.5.253 10.12968/hmed.2018.79.5.253 PMC5963675 29727228

[15] He C, He W, Hou J et al (2020) Bone and muscle crosstalk in aging. Front Cell Dev Biol 8. 10.3389/fcell.2020.585644 10.3389/fcell.2020.585644 PMC7758235 33363144

[16] Aromaa A, Koskinen S ed (2004) Health and functional capacity in Finland. Baseline results of the Health 2000 Health Examination Survey. Helsinki: Publications of the National Public Health Institute B12

[17] Heistaro S ed (2008) Methodology report: Health 2000 Survey. Helsinki: Publications of the National Public Health Institute B26

[18] Borodulin K, Harald K, Jousilahti P et al (2016) Time trends in physical activity from 1982 to 2012 in Finland. Scand J Med Sci Sports 26(1):93–100. 10.1111/SMS.12401 25559167 10.1111/sms.12401

[19] Yearbook of Alcohol and Drug Statistics (2003) Helsinki: National Research and Development Centre for Welfare and Health [STAKES]

[20] Paalanen L, Männistö S, Virtanen MJ et al (2006) Validity of a food frequency questionnaire varied by age and body mass index. J Clin Epidemiol 59(9):994–1001. 10.1016/J.JCLINEPI.2006.01.002 16895824 10.1016/j.jclinepi.2006.01.002

[21] Reinivuo H, Hirvonen T, Ovaskainen ML et al (2010) Dietary survey methodology of FINDIET 2007 with a risk assessment perspective. Public Health Nutr 13(6A):915–919. 10.1017/S1368980010001096 20513260 10.1017/S1368980010001096

[22] Sainio P, Koskinen S, Heliövaara M et al (2006) Self-reported and test-based mobility limitations in a representative sample of Finns aged 30+. Scand J Public Health 34(4):378–386. 10.1080/14034940500489859 16861188 10.1080/14034940500489859

[23] Folstein MF, Folstein SE, McHugh PR (1975) “Mini-mental state”. A practical method for grading the cognitive state of patients for the clinician. J Psychiatr Res 12(3):189–198. 10.1016/0022-3956(75)90026-6 10.1016/0022-3956(75)90026-6 1202204

[24] Nielsen BR, Abdulla J, Andersen HE et al (2018) Sarcopenia and osteoporosis in older people: a systematic review and meta-analysis. Eur Geriatr Med 9(4):419–434. 10.1007/S41999-018-0079-6 34674498 10.1007/s41999-018-0079-6

[25] Looker AC, Frenk SM (2015) Percentage of adults aged 65 and over with osteoporosis or low bone mass at the femur neck or lumbar spine: United States, 2005–2010. Health E-Stat, NCHS. Available online: https://www.cdc.gov/nchs/data/hestat/osteoporsis/osteoporosis2005_2010.pdf. Accessed 21.8.2024

[26] Kanis JA et al (2000) Risk of hip fracture according to the World Health Organization criteria for osteopenia and osteoporosis. Bone 12(5):585–590. 10.1016/S8756-3282(00)00381-1 10.1016/s8756-3282(00)00381-1 11062343

[27] López-Rodríguez F, Mezquita-Raya P, de Dios LJ et al (2003) Performance of quantitative ultrasound in the discrimination of prevalent osteoporotic fractures in a bone metabolic unit. Bone 32(5):571–578. 10.1016/s8756-3282(03)00058-9 12753874 10.1016/s8756-3282(03)00058-9

[28] Cruz-Jentoft AJ, Gonzalez MC, Prado CM (2023) Sarcopenia ≠ low muscle mass. Eur Geriatr Med 1:1–4. 10.1007/S41999-023-00760-7/FIGURES/1 10.1007/s41999-023-00760-7 36869279

[29] Huo YR, Suriyaarachchi P, Gomez F et al (2015) Phenotype of osteosarcopenia in older individuals with a history of falling. J Am Med Dir Assoc 16(4):290–295. 10.1016/j.jamda.2014.10.018 25512216 10.1016/j.jamda.2014.10.018

[30] Okamura H, Ishikawa K, Kudo Y et al (2020) Risk factors predicting osteosarcopenia in postmenopausal women with osteoporosis: a retrospective study. PLoS One 15(8). 10.1371/JOURNAL.PONE.0237454 10.1371/journal.pone.0237454 PMC7413553 32764814

[31] Reiss J, Iglseder B, Alzner R et al (2019) Sarcopenia and osteoporosis are interrelated in geriatric inpatients. Z Gerontol Geriatr 52(7):688. 10.1007/S00391-019-01553-Z 31049683 10.1007/s00391-019-01553-z PMC6817738

