# Correction: Osteosarcopenia in Finland: prevalence and associated factors

**DOI:** 10.1007/s11657-026-01714-9

**Published:** 2026-06-16

**Authors:** Matias Blomqvist, Maria Nuotio, Katri Sääksjärvi, Seppo Koskinen, Sari Stenholm

**Affiliations:** 1 University of Turku, Turun Yliopisto, Turku, Finland; 2 Department of Geriatric Medicine, University of Turku, Turku, Finland; 3 Department of Geriatric Medicine, Turku University Hospital and University of Turku, Turku, Finland; 4 Population Health Unit, Finnish Institute for Health and Welfare, Helsinki, Finland; 5 Department of Public Health, University of Turku and Turku University Hospital, Turku, Finland; 6 Centre for Population Health Research, University of Turku and Turku University Hospital, Turku, Finland; 7 Research Services, Turku University Hospital and University of Turku, Turku, Finland

**Correction: Archives of Osteoporosis (2024) 19:80**

10.1007/s11657-024-01439-7

The original online version of this article was revised:

In the original version of this article, 364 participants were inadvertently excluded from the “no sarcopenia, no osteoporosis” reference group, resulting in an incorrect sample size of 1602 instead of the correct 1966.

All analyses have now been repeated using the corrected dataset. The overall conclusions of the manuscript remain unchanged. However, the correction led to minor changes in statistical estimates and significance for several variables, primarily those with borderline p-values in the original analysis.

The specific changes to Table 3 (“Association of sociodemographic and lifestyle factors, and physical and mental conditions with osteosarcopenia group”) are as follows:

Meal frequency (infrequent meals < 1–2 per day vs. frequent meals):

Originally reported as statistically significant in the osteoporosis-only group (OR 0.65, 95% CI 0.42–0.99), this association is no longer statistically significant in the corrected analysis (OR 0.67, 95% CI 0.45–1.02).

Short MMSE:

Originally reported as non-significant (13.3 [12.9–13.6] vs. 13.6 [13.5–13.7]), the corrected analysis shows borderline significantly lower adjusted MMSE scores in the osteoporosis-only group (13.2 [12.9–13.5] vs. 13.6 [13.5–13.7]).

Diabetes (sarcopenia-only group):

Originally non-significant (OR 1.33, 95% CI 0.86–2.05), now significantly associated with higher odds compared to the reference group (OR 1.59, 95% CI 1.05–2.39).

Cardiovascular disease or heart failure (sarcopenia-only group):

Originally non-significant (OR 1.36, 95% CI 0.98–1.89), now significantly associated (OR 1.41, 95% CI 1.03–2.94).

Stroke (sarcopenia-only group):

Originally borderline non-significant (OR 1.60, 95% CI 1.00–2.56), now statistically significant (OR 1.66, 95% CI 1.04–2.63).

Oral health other than good (sarcopenia-only group):

Originally non-significant (OR 1.43, 95% CI 0.96–2.13), now statistically significant (OR 1.48, 95% CI 1.02–2.15).

The original article has been corrected.

