# Supplementary material for: Osteosarcopenia in Finland: prevalence and associated factors
Source: Arch Osteoporos. 2024 Aug 30;19(1):80. doi: 10.1007/s11657-024-01439-7 (PMC11364597; doi:10.1007/s11657-024-01439-7)
Supplement: Supplementary file 1 — Supplementary file1 (PDF 566 KB) [file 11657_2024_1439_MOESM1_ESM.pdf]

# Supplement 1: Extended methods section.

## Study population

This study is based on the Health 2000 survey, a nationwide health examination survey collecting information on the health, functional capacity and well-being of the Finnish population in 2000-2001. Ten thousand people aged 18 and older were selected randomly from the national population register, using a stratified two-stage cluster sampling design. To obtain a sufficient number of observations from the older population, the sampling fraction for people aged 80 and older was doubled. Community-dwelling as well as institutionalized people living in mainland Finland were included. The implementation of the Health 2000 survey has been reported elsewhere in more detail (Aromaa et al., 2004). The study sample included 8028 subjects, of which 3439 were 55 years old or older. For this study, we included participants aged 55 years or older from whom information on sarcopenia and osteoporosis was available (n=2506, 72.9 %).

## Anthropometric measurements

Weight was either measured with a bioimpedance measurement device InBody 3.0 (n=2185, 87.2%), a digital scale (n=122, 4.9%) or self-reported (n=196, 7.8%) if measurement was not available. Weight information was missing for 3 subjects. Height was measured for 2103 (83.9%) subjects and self-reported by 184 (7.3%) subjects. Height information was missing for 219 (8.7%) subjects. Body Mass Index was calculated as weight in kilograms divided by height in meters squared and further classified as underweight (<18.5), normal weight (18.5 – 24.9), overweight (25 – 29.9) and obese (≥30).

## Sociodemographic and lifestyle factors

Sociodemographic factors included age, sex, level of education (low, intermediate and hight) and marital status (married, cohabiting, or living alone).

Information on lifestyle factors were obtained from interview or survey questionnaire. Level of physical activity was self-assessed on a 3-level scale (exercise training “Leisure time includes strenuous physical exercise at least 3 hours per week”, active “Leisure time includes walking, bicycling and other forms of physical activity at least 4 hours per week” or inactive “Leisure time consists of reading, television, or activities not involving physical activity”)(Borodulin et al., 2016).

Smoking status was categorized as current/past or never.

Alcohol use was surveyed and categorized into three levels: abstinence, low-to-medium use (less than 139 g/week for women, less than 279 g/week for men), and high use (140 g/week or more for women, 280 g/week or more for men) (Yearbook of Alcohol and Drug Statistics. Helsinki, Finland: National Research and Development Centre for Welfare and Health [STAKES]; 2003).

Dietary habits were assessed using a validated semi-quantitative 128-item food frequency questionnaire (FFQ) (Paalanen et al., 2006) and the food consumption and nutrient intakes were calculated using the national food composition database (Fineli®) and in-house software (Reinivuo et al., 2010). The foods were categorized as follows: dairy products, grain products, bread spreads, vegetable based foods, potato, pasta and rice, meat based foods, fish based foods, fruits and berries, desserts, candy and snacks, and drinks. Subjects were asked to estimate the frequency of consumption for every item on the following scale: never or rarely, 1-3 times per month, once a week, 2-4 times per week, 5-6 times per week, once daily, 2-3 times daily, 4-5 times daily, and 6 or more times daily. Portion size was estimated relative to exemplary pictures of portion sizes. Variables included in this study were dairy product intake (g/d), protein intake (g/d), and total

calcium intake from diet and supplements (g/d). The FFQ contained also additional questions about meal patterns and special diets, thus, variables on meal frequency (infrequent meals [fewer than 1-2 per day]; frequent meals [at least 1-2 times per day]), and lactose free diet (yes; no) were included. Validity of all completed questionnaires was evaluated by a nutritionist. The short-term repeatability of the questionnaire was validated by having 209 randomly selected subjects repeat the survey after 8 months. 180 Of these subjects completed the repeated frequency questionnaire. The intraclass correlation coefficient for foods ranged between 0.16 and 0.82, and for nutrient intakes the figures were between 0.22 and 0.72. The frequency questionnaire was validated by having randomly selected 470 subjects keep food diary for three days. 294 (76 %) of the randomly selected subjects completed a valid diary and a valid frequency questionnaire.

Anthropometric measurements included body mass index (BMI) based on body weight and height measurements, calculated as $kg/m^2$, and self-reported weight loss during the past 12 months.

## Physical and mental function

At the health examination, maximal gait speed was measured over 6.1 meter course (Sainio et al., 2006). Subjects were asked to walk the distance as quickly as they could, starting from their normal, standing posture behind the start line and continuing at full speed beyond the end line. They were allowed to use walking aids.

Grip strength was measured using an electronic device (Good Strength, IGS01, Metitur Oy, Finland) while the subject was sitting and resting their elbow on a table, gripping the handle of the device in a neutral position (slight dorsiflexion). Height of the chair was adjusted so that the angle of the elbow joint was approximately 110 degrees. The dominant hand was used, while the other hand was resting on the table or on the lap. The grip size was adjusted so that the proximal interphalangeal joint of the index finger was 90 degrees and the participant felt comfortable with

grip size. Measurement was repeated after 30 seconds, and if there was a difference greater than 10%, a third measurement was taken. The highest result was ultimately used for analysis.

In addition, subjects were asked “Can you walk 0.5 km without resting?” and “Can you climb up one flight of stairs without resting?”. Subjects were considered to have mobility limitation if they reported any difficulties in walking 500 m or stair climbing.

Similarly, performance in activities of daily living (ADL) were asked “How do you manage following activities (getting on or off the bed, getting clothes on or off, showering or using the bathroom)?”

Abbreviated version of the 30 point Mini-Mental State Examination, with a maximum score of 16 points was used to assess memory impairment (Folstein et al., 1975). The following were included from the orignial: Three 1-point questions on orientation. A 3-point objective to repeat a list of three words, and a 3-point objective to recall it after another objective. A 5-point objective to count down from 100 in decrements of 7. A 1-point objective to repeat a spoken sentence. A 1-point objective to copy a drawing of two overlapping shapes.

Subject’s mood was concidered depressed if they reported ”feeling mostly sad or depressed at the day of the interview.

## Chronic conditions

Information on chronic conditions was based on self-reported diagnoses. Disease categories used in this study were diabetes, cancer excluding non-melanoma skin cancer, ocular disease (cataract, glaucoma, or retinal degeneration), hearing loss (hearing impairment of any type), psychiatric illness, arthrosis of the knee or the hip, pulmonary disease (asthma, chronic obstructive pulmonary disease, or chronic bronchitis), CVD or heart failure (myocardial infarction, angina pectoris, or heart failure), and stroke. Participants were also asked to evaluate their oral health

condition and they were grouped as ”good” and “other than good” (including somewhat good, average, somewhat poor and poor).

## Laboratory test

Fasting serum and spot urine samples were collected at the health examinations using standard procedures. The samples were immediately frozen to –20°C on site, packed in dry ice and transferred to the final storage location (-70 °C) before use.

Serum vitamin-D-25 was determined by radioimmunoassay (RIA, Diasorin, Minnesota).

GHbA1c was determined with an immunoturbidimetric method (Hemoglobin A1c assay; Abbott Laboratories).

Plasma creatinine (µmol/l) was analyzed by an enzymatic method (Abbott Laboratories, Abbott Park, IL, USA) from the frozen plasma samples and the detection limit was 8.8 µmol/l.

Urine albumin (mg/l) was measured by an immunoturbidimetric method (Abbott Laboratories, Abbott Park, IL, USA).

Serum testosterone was assessed from males only, and Chemiluminescent Microparticle Immunoassay (CMIA) method was used (Abbott Laboratories, Abbott Park, IL, USA).

C-reactive protein (CRP) was analyzed by an automated analyzer (Optima; Thermo Electron Oy, Vantaa, Finland) and an ultrasensitive immunoturbidimetric test (Ultrasensitive CRP; Orion Diagnostica, Espoo, Finland), with a detection limit of 0.20 mg/l.

For FEV1, Vitalograph bellow spirometers (Vitalograph Ltd., Buckingham, UK) were used to measure lung function. Highest readings from at least two valid measurements were used, in accordance with the guidelines (Crapo et al., 1994)

## References


Aromaa A, Koskinen S ed (2004) Health and functional capacity in Finland. Baseline Results of the Health 2000 Health Examination Survey. Helsinki: Publications of the National Public Health Institute B12

Borodulin, K., Harald, K., Jousilahti, P., Laatikainen, T., Männistö, S., & Vartiainen, E. (2016). Time trends in physical activity from 1982 to 2012 in Finland. *Scandinavian Journal of Medicine & Science in Sports*, *26*(1), 93–100. https://doi.org/10.1111/SMS.12401

Paalanen, L., Männistö, S., Virtanen, M. J., Knekt, P., Räsänen, L., Montonen, J., & Pietinen, P. (2006). Validity of a food frequency questionnaire varied by age and body mass index. *Journal of Clinical Epidemiology*, *59*(9), 994–1001. https://doi.org/10.1016/J.JCLINEPI.2006.01.002

Reinivuo, H., Hirvonen, T., Ovaskainen, M. L., Korhonen, T., & Valsta, L. M. (2010). Dietary survey methodology of FINDIET 2007 with a risk assessment perspective. *Public Health Nutrition*, *13*(6A), 915–919. https://doi.org/10.1017/S1368980010001096

Sainio, P., Koskinen, S., Heliövaara, M., Martelin, T., Härkänen, T., Aromaa, A., Hurri, H., & Miilunpalo, S. (2006). Self-reported and test-based mobility limitations in a representative sample of Finns aged 30+. *Scandinavian Journal of Public Health*, *34*(4), 378–386. https://doi.org/10.1080/14034940500489859

Folstein MF, Folstein SE, McHugh PR. “Mini-mental state”. A practical method for grading the cognitive state of patients for the clinician. *J Psychiatr Res*. 1975;12(3):189-198. doi:10.1016/0022-3956(75)90026-6

Crapo O, Hankinson JL, Irvin C, et al. Standardization of Spirometry, 1994 Update. American Thoracic Society. *Am J Respir Crit Care Med*. 1995;152(3):1107-1136. doi:10.1164/AJRCCM.152.3.7663792
