# Supplementary material for: Osteosarcopenia in Finland: prevalence and associated factors
Source: Arch Osteoporos. 2024 Aug 30;19(1):80. doi: 10.1007/s11657-024-01439-7 (PMC11364597; doi:10.1007/s11657-024-01439-7)
Supplement: Supplementary file 2 — Supplementary file2 (PDF 368 KB) [file 11657_2024_1439_MOESM2_ESM.pdf]

Supplement 2.

Table S1: The association of sociodemographic, lifestyle factors, physical and mental conditions with osteosarcopenic groups, osteosarcopenic group used as reference.

	Osteosarcopenia	*Probable sarcopenia only*	*Osteoporosis only*	*No sarcopenia, no osteoporosis*
Sosiodemographic factors	*OR. Reference*	*OR (95% CI)*	*OR (95% CI)*	*OR (95% CI)*
Married or cohabiting vs. Living alone	1.00	2.51 (1.23 – 5.10)	3.53 (1.83 – 6.80)[b]	3.67 (2.00 – 6.74)[b]
Lifestyle factors				
Physical activity				
Exercise training vs. Inactive	1.00	1.63 (0.37 – 7.11)	4.25 (1.04 – 17.41)	4.49 (1.21 – 16.62)
Active vs. Inactive	1.00	1.66 (0.98 – 2.81)	2.39 (1.31 – 4.35)	3.01 (1.82 – 4.99)[b]
Alcohol				
High use vs. Abstinence	1.00	0.29 (0.06 – 1.52)	0.92 (0.18 – 4.84)	0.30 (0.07 – 1.28)
Medium or low use vs. Abstinence	1.00	0.97 (0.55 – 1.72)	1.14 (0.67 – 1.94)	1.42 (0.87 – 2.32)
Infrequent meals (fewer than 1-2 per day) vs. frequent meals	1.00	0.78 (0.46 – 1.33)	0.67 (0.36 – 1.26)	1.00 (0.59 – 1.70)
Lactose free diet	1.00	0.64 (0.25 – 1.59)	1.43 (0.62 – 3.32)	0.75 (0.35 – 1.57)
Anthropometric measurements				
Body mass index (kg/m²)				
Underweight (<18.5) vs. Normal weight (18.5-25)	1.00	0.46 (0.14 – 1.59)	0.44 (0.11 – 1.76)	0.10 (0.02 – 0.38) [b]
Overweight (25 – 29.9) vs. Normal weight (18.5-25)	1.00	1.56 (0.92 – 2.66)	1.53 (0.88 – 2.65)	2.56 (1.59 – 4.20)[b]
Obese (≥30) vs. Normal weight (18.5-25)	1.00	2.81 (1.51 – 5.23)[b]	1.49 (0.76 – 2.92)	3.16 (1.75 – 5.71)[b]
Weight loss				
>5kg unintentionally vs. No weight loss	1.00	2.29 (0.86 – 6.08)	1.84 (0.61 – 5.57)	1.19 (0.48 – 3.00)
>5kg intentionally vs. No weight loss	1.00	2.33 (0.30 – 18.00)	2.13 (0.21 – 21.83)	1.89 (0.20 – 17.84)
1-5 kg vs. No weight loss	1.00	1.28 (0.64 – 2.53)	0.94 (0.45 – 1.96)	1.04 (0.55 – 1.95)
Physical and mental function				
Mobility limitation	1.00	0.46 (0.24 – 0.87)	0.31 (0.16 – 0.62)[b]	0.16 (0.09 – 0.28)[b]
ADL limitation	1.00	0.56 (0.33 – 0.94)	0.33 (0.19 – 0.56)[b]	0.13 (0.08 – 0.20)[b]
Depressive mood	1.00	0.64 (0.38 – 1.07)	0.37 (0.21 – 0.62)[b]	0.36 (0.22 – 0.59)[b]
Chronic conditions				

Diabetes	1.00	1.67 (0.82 – 3.40)	0.95 (0.36 – 2.47)	1.05 (0.53 – 2.08)
Cancer excluding non-melanoma skin cancer	1.00	1.45 (0.69 – 3.05)	2.21 (1.04 – 4.72)	1.74 (0.90 – 3.39)
Ocular disease	1.00	1.26 (0.74 – 2.16)	0.66 (0.37 – 1.16)	0.75 (0.47 – 1.20)
Hearing loss	1.00	0.79 (0.46 – 1.36)	1.06 (0.60 – 1.88)	0.96 (0.57 – 1.62)
Psychiatric illness	1.00	0.61 (0.32 – 1.18)	0.44 (0.21 – 0.92)	0.41 (0.24 – 0.72)
Arthrosis of the knee or the hip	1.00	0.91 (0.54 – 1.56)	0.89 (0.54 – 1.47)	0.58 (0.38 – 0.88)
Pulmonary disease	1.00	0.81 (0.46 – 1.43)	1.10 (0.56 – 2.16)	0.57 (0.33 – 0.98)
CVD or heartfailure	1.00	0.76 (0.46 – 1.25)	0.62 (0.35 – 1.10)	0.53 (0.35 – 0.82)
Stroke	1.00	0.97 (0.41 – 2.28)	0.81 (0.30 – 2.14)	0.59 (0.27 – 1.27)
Oral health other than good	1.00	0.87 (0.50 – 1.50)	0.55 (0.29 – 1.06)	0.59 (0.34 – 1.01)

Notes: Analyses are adjusted for age, sex, smoking and education. Results are presented for categorical variables as odds ratios (OR) and mean values for continues variables, and their 95% confidence intervals (CI). Analyses are adjusted for age and sex. [b]Statistically significant difference compared to the no sarcopenia, no osteoporosis group after Bonferroni correction.

Table S2:

The association of sociodemographic and lifestyle factors, and physical and mental conditions with osteosarcopenia group. Analysis adjusted for age and sex

	No sarcopenia, no osteoporosis	*Sarcopenia-only*	*Osteoporosis-only*	*Osteosarcopenia*
Sosiodemographic factors	*OR. Reference*	*OR (95% CI)*	*OR (95% CI)*	*OR (95% CI)*
Married or cohabiting vs. Living alone	1.00	0.67 (0.49 - 0.91)	0.97 (0.68 - 1.37)	0.28 (0.16 - 0.51)[b]
Lifestyle factors				
Physical activity				
Exercise training vs. Inactive	1.00	0.35 (0.18 - 0.67)[b]	1.00 (0.55 - 1.83)	0.24 (0.06 - 0.87)[b]
Active vs. Inactive	1.00	0.54 (0.40 - 0.73)[b]	0.82 (0.57 - 1.18)	0.34 (0.21 - 0.56)[b]
Alcohol				
High use vs. Abstinence	1.00	1.13 (0.45 - 2.86)	3.45 (1.49 – 7.83)	4.07 (0.89 - 18.63)
Medium or low use vs. Abstinence	1.00	0.72 (0.52 – 1.00)	0.82 (0.59 - 1.15)	0.74 (0.46 - 1.18)
Infrequent meals (fewer than 1-2 per day) vs. frequent meals	1.00	0.81 (0.56 – 1.19)	0.70 (0.46 – 1.04)	1.05 (0.62 – 1.79)
Lactose free diet	1.00	0.89 (0.47 - 1.71)	1.87 (1.12 - 3.13)	1.34 (0.65 – 2.75)
	Adjusted mean (95% CI)	*Adjusted mean (95% CI)*	*Adjusted mean (95% CI)*	*Adjusted mean (95% CI)*
Dairy product intake. g/d	635 (618 - 652)	639 (584 - 693)	587 (528 - 646)	525 (449 - 601)
Protein intake. g/d	95 (93 - 97)	99 (94 - 105)	91 (85 - 97)	83 (75 - 90)
Calcium intake. mg/d	1452 (1379 - 1526)	1453 (1246 - 1660)	1499 (1307 - 1689)	1353 (1091 - 1614)
Anthropometric measurements				
Body mass index (kg/m²)	*OR. Reference*	*OR (95% CI)*	*OR (95% CI)*	*OR (95% CI)*
Underweight (<18.5) vs. Normal weight (18.5-25)	1.00	3.95 (0.93 – 16.77)	3.98 (0.98 – 16.20)	9.85 (2.70 – 35.96)[b]
Overweight (25 – 29.9) vs. Normal weight (18.5-25)	1.00	0.59 (0.40 - 0.86)	0.59 (0.40 - 0.86)	0.40 (0.25 - 0.65)[b]
Obese (≥30) vs. Normal weight (18.5-25)	1.00	0.91 (0.63 - 1.30)	0.47 (0.30 - 0.71)[b]	0.33 (0.18 - 0.58)[b]
Weight loss				
>5kg unintentionally vs. No weight loss	1.00	1.97 (1.12 - 3.44)	1.52 (0.77 – 3.00)	0.82 (0.34 - 1.99)
>5kg intentionally vs. No weight loss	1.00	1.26 (0.53 – 3.01)	1.11 (0.47 - 2.62)	0.55 (0.06 – 5.00)
1-5 kg vs. No weight loss	1.00	1.21 (0.81 - 1.81)	0.91 (0.57 - 1.46)	0.99 (0.54 - 1.82)
Physical and mental function	*OR. Reference*	*OR (95% CI)*	*OR (95% CI)*	*OR (95% CI)*
Mobility limitation	1.00	3.11 (2.23 - 4.32)[b]	2.00 (1.43 – 2.81)[b]	6.73 (3.77 - 12.04)[b]
ADL limitation	1.00	4.59 (3.37 – 6.24)[b]	2.57 (1.75 - 3.78)[b]	8.21 (5.13 - 13.14)[b]

Depressive mood	1.00	1.54 (1.03 - 2.31)[b]	1.06 (0.62 - 1.79)	2.45 (1.41 - 4.24)[b]
	Adjusted mean (95% CI)	*Adjusted mean (95% CI)*	*Adjusted mean (95% CI)*	*Adjusted mean (95% CI)*
Grip strength. kg	34.0 (33.7 - 34.3)	20.1 (19.3 - 20.9) [b]	32.7 (31.8 - 33.6)	23.2 (22.0 - 24.4) [b]
Gait speed. m/s	1.53 (1.51 - 1.54)	1.27 (1.22 - 1.31) [b]	1.43 (1.38 - 1.48) [b]	1.22 (1.15 - 1.28) [b]
FEV1 spirometry. L	2.54 (2.52 - 2.57)	2.36 (2.28 - 2.44) [b]	2.57 (2.48 - 2.66)	2.36 (2.24 - 2.49)
Short MMSE	13.6 (13.5 - 13.7)	12.9 (12.5 - 13.2) [b]	13.2 (12.9 - 13.6)	12.6 (12.2 - 13.1) [b]
Chronic conditions	*OR. Reference*	*OR (95% CI)*	*OR (95% CI)*	*OR (95% CI)*
Diabetes	1.00	1.59 (1.06 - 2.40)	0.85 (0.46 - 1.57)	0.88 (0.45 - 1.72)
Cancer excluding non-melanoma skin cancer	1.00	0.84 (0.50 - 1.40)	1.29 (0.83 – 2.02)	0.58 (0.30 - 1.12)
Ocular disease	1.00	1.71 (1.22 - 2.39)	0.91 (0.62 - 1.33)	1.31 (0.82 – 2.10)
Hearing loss	1.00	0.82 (0.58 - 1.16)	1.12 (0.78 - 1.61)	1.02 (0.61 - 1.71)
Psychiatric illness	1.00	1.54 (1.03 - 2.31)	1.06 (0.62 - 1.79)	2.45 (1.41 - 4.24)
Arthrosis of the knee or the hip	1.00	1.56 (1.16 - 2.10)	1.51 (1.09 - 2.10)	1.73 (1.14 - 2.62)
Pulmonary disease	1.00	1.46 (1.02 - 2.10)	1.96 (1.36 - 2.85)[b]	1.88 (1.10 - 3.19)
CVD or heartfailure	1.00	1.43 (1.04 - 1.96)	1.22 (0.83 - 1.80)	1.91 (1.25 - 2.91)
Stroke	1.00	1.70 (1.07 - 2.70)	1.37 (0.71 - 2.63)	1.70 (0.78 - 3.68)
Oral health other than good	1.00	1.50 (1.03 - 2.19)	0.92 (0.58 - 1.47)	1.67 (0.97 - 2.87)
Laboratory biomarkers	*Adjusted mean (95% CI)*	*Adjusted mean (95% CI)*	*Adjusted mean (95% CI)*	*Adjusted mean (95% CI)*
Vitamin D-25. nmol/l	47.3 (46.5 - 48.1)	43.4 (41.0 - 45.8)	47.9 (45.1 - 50.7)	40.9 (37.2 - 44.6)
GHbA1c. %	5.60 (5.56 - 5.63)	5.66 (5.56 - 5.76)	5.47 (5.36 - 5.59)	5.46 (5.31 - 5.61)
Creatinine. µmol/l	77.2 (76.4 - 77.9)	77.1 (74.8 - 79.3)	76.9 (74.3 - 79.5)	73.0 (69.6 - 76.4)
Urine albumin. Log(mg/l)	2.46 (2.38 - 2.53)	2.67 (2.48 - 2.87)	2.64 (2.41 - 2.88)	2.33 (2.06 - 2.61)
Testosterone (males). nmol/l	15.2 (14.8 - 15.6)	15.9 (14.5 - 17.3)	18.2 (15.5 - 21.0)	12.3 (8.6 - 16.0)
CRP. Log(mg/l)	0.14 (0.07 - 0.20)	0.62 (0.43 - 0.81)	0.13 (-0.08 - 0.35)	0.31 (0.03 - 0.60)

Notes: Results are presented for categorical variables as odds ratios (OR) and mean values for continues variables, and their 95% confidence intervals (CI). Analyses are adjusted for age and sex. [b] Statistically significant difference compared to no sarcopenia, no osteoporosis group after Bonferroni correction.
